# Recognizing Sights, Smells, and Sounds with Gnostic Fields

**DOI:** 10.1371/journal.pone.0054088

**Published:** 2013-01-24

**Authors:** Christopher Kanan

**Affiliations:** Department of Computer Science and Engineering, University of California San Diego, La Jolla, California, United States of America; Wake Forest School of Medicine, United States of America

## Abstract

Mammals rely on vision, audition, and olfaction to remotely sense stimuli in their environment. Determining how the mammalian brain uses this sensory information to recognize objects has been one of the major goals of psychology and neuroscience. Likewise, researchers in computer vision, machine audition, and machine olfaction have endeavored to discover good algorithms for stimulus classification. Almost 50 years ago, the neuroscientist Jerzy Konorski proposed a theoretical model in his final monograph in which competing sets of “gnostic” neurons sitting atop sensory processing hierarchies enabled stimuli to be robustly categorized, despite variations in their presentation. Much of what Konorski hypothesized has been remarkably accurate, and neurons with gnostic-like properties have been discovered in visual, aural, and olfactory brain regions. Surprisingly, there have not been any attempts to directly transform his theoretical model into a computational one. Here, I describe the first computational implementation of Konorski's theory. The model is not domain specific, and it surpasses the best machine learning algorithms on challenging image, music, and olfactory classification tasks, while also being simpler. My results suggest that criticisms of exemplar-based models of object recognition as being computationally intractable due to limited neural resources are unfounded.

## Introduction

We can recognize thousands of object categories using our senses [1]. While our ability to quickly do this seems effortless, computer scientists have yet to construct algorithms that rival our capabilities [1]. The best algorithms are often domain specific and combine many types of engineered features. But while computer scientists have only been working on these problems since the 1960s, our brains have been forged by evolution over millions of years. Our ancestors needed to remotely recognize stimuli using vision, audition, and olfaction to find food, identify mates, and cope with predators. To do these tasks, the mammalian brain hierarchically processes sensory information, enabling stimuli to be classified into general categories despite non-relevant stimulus variation. For example, we can recognize our mother's face from others despite changes in viewpoint, distinguish between the voices of our friends when they are shouting or whispering, and identify the scent of a mango even as the intensity of its odor varies as it ripens.

In his final monograph, the theoretical neurobiologist Jerzy Konorski developed a rich theory for how the brain accomplishes invariant stimulus recognition across sensory modalities, including olfaction, vision, audition, and gustation [2]. I call his proposal Gnostic Field Theory. Konorski hypothesized that an object category is represented in the brain by a redundant set (files) of gnostic neurons (units), which sit near the top of a sensory processing hierarchy for a given modality. Each gnostic neuron is tuned to a complex stimulus-pattern from a particular category. A gnostic set contains a population of gnostic units all tuned to recognize the same category. Gnostic fields are populations of competing gnostic sets, which enable discrimination among categories.

Gnostic neurons have been claimed to be similar to Lettvin's grandmother cells (e.g., [3], [4]) and both are “localist” representations. However, there are notable differences between the two theories. In grandmother cell theory, only a single neuron sitting on top of a sensory processing hierarchy categorizes a particular object class, and this neuron is only active when it detects a pattern consistent with the object class it is tuned to recognize [4]. It is important to note that this definition is not universally agreed upon, and some define grandmother cells to be more similar to gnostic units, e.g., [5]. Gnostic Field Theory posits a *redundant* population of gnostic neurons exists near the top of a sensory processing hierarchy, which are most active when exposed to stimuli from the category they represent. They may still exhibit attenuated activity when exposed to stimuli from other categories, and Konorski states that when trying to categorize an unfamiliar stimulus into a known category the activity of the entire gnostic field will increase. However, gnostic neurons alone are not sufficient to enable robust categorization. The population of gnostic neurons representing a category are organized into a gnostic set, and gnostic sets act as competing sub-networks within a gnostic field [2]. Although there was no electrophysiological evidence for gnostic neurons when they were first proposed, neurons with similar properties have since been discovered in the visual [6], [7], aural [8], [9], and olfactory [10] systems.

The neural mechanisms used to classify stimuli have been most studied in the primate visual system, especially the mechanisms used by the ventral “object recognition” pathway from primary visual cortex (V1) to inferior temporal cortex (IT). The standard model is a hierarchy of increasingly complex representations [2], [11] beginning with simple cells in V1 that respond to edges and bars. As predicted by Konorski [2], [3], IT contains neurons tuned to views of specific objects [6], [7] and there is evidence of neurons with similar properties in medial temporal lobe (MTL) [12], [13]. These neurons respond vigorously to specific object categories and many are tolerant to changes in appearance, scale, and location in the visual field. Most exhibit an attenuated response to other stimuli, ruling out grandmother cell coding, but not gnostic neuron coding. In humans, gnostic fields for faces, places, and tools have been discovered using functional imaging (see [14] for a review), in largely the same locations Konorski predicted.

Olfactory and aural stimuli are also processed by a hierarchy of brain regions, with gnostic-like activity at the top levels. For sounds, both conspecific “call detector” neurons [8] and neurons responding to the vocal signatures of familiar individuals have been found in monkey prefrontal cortex [9]. Functional neuroimaging of humans places the gnostic field for recognizing familiar odors in piriform cortex [15], and rat piriform neurons exhibit activity consistent with gnostic neurons [10].

**Figure 1:**
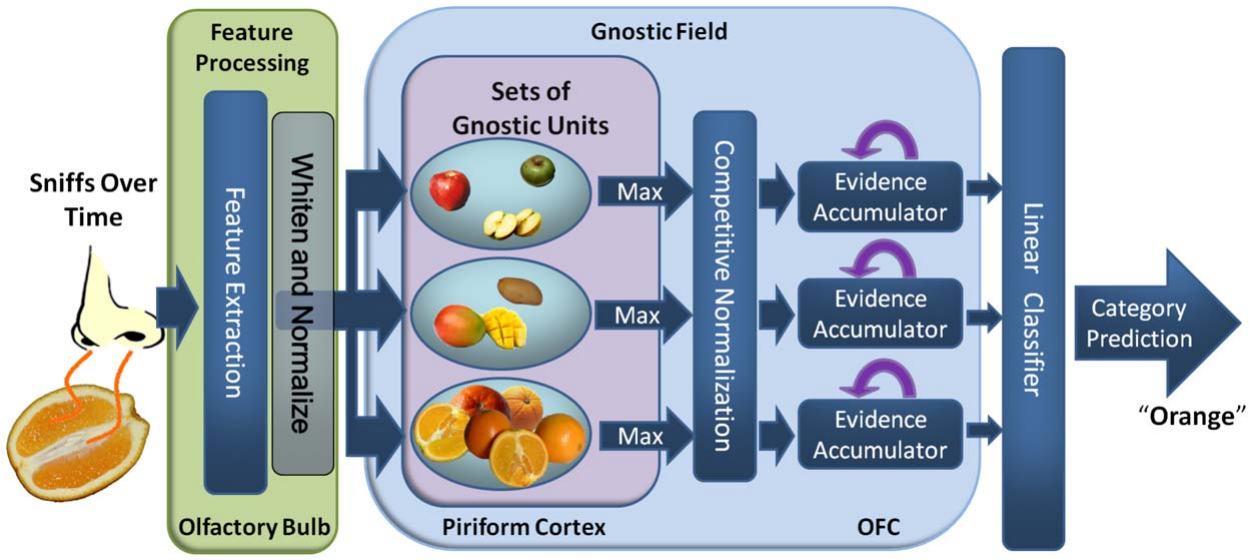
Example olfactory gnostic field. A high-level depiction of the model presented in this paper applied to classifying apples, mangos, and oranges using smell. The olfactory features are acquired over time, and at each time step they are decorrelated and whitened to normalize the signal's variance. These feature processing steps are hypothesized to occur in the olfactory bulb. Temporally local olfactory processing occurs in the gnostic sets for each category, with some of the units in the set for oranges responding strongest. The most active unit in each set serves as its output. Competitive normalization is used to adjust the activity of the gnostic sets, resulting in the output of the mango and apple sets being suppressed. For olfactory processing, the gnostic sets would likely be located in piriform cortex [15]. Evidence acquired by the gnostic sets is then accumulated across time, analogous to the processing in orbitofrontal cortex (OFC) [24]. Finally, the evidence from all categories is combined using a linear classifier. Similar example systems can be constructed for other sensory modalities, which would employ different brain regions. Note that only a single channel is depicted, but the experiments with visual data used three high-dimensional channels with differing chromatic and luminance properties (see text for details).

Here, I develop the first computational implementation of Konorski's universal theory of recognition across sensory modalities, enabling its effectiveness to be evaluated. I apply the model to large many-category recognition tasks using publicly available image, sound, and odor data sets. The same model can be used for both categorization and identification. From a computational perspective, the model is simple, and the architecture could conceivably be implemented by a biological nervous system. A high-level schematic of an olfactory gnostic field for discriminating among apples, mangos, and oranges is presented in Fig. 1.

The inputs to a gnostic field are high-dimensional features that may have been segregated into multiple channels, e.g., for vision they consist of oriented edge and bar detectors from luminance and opponent-color channels. Each channel's features are normalized using a whitening transformation [16], a form of decorrelation approximately performed by early visual [17], aural [18], and olfactory sensory systems [19]. These normalized features project to a bank of gnostic sets. There is one gnostic set per category and channel combination, and each gnostic set processes data either spatially or temporally locally. The units in a gnostic set act as pattern detectors for category-specific features from across the visual field or over time for sounds and odors. These units are most active when they recognize their input as belonging to their category. The output of a gnostic set is given by the unit in the set with the largest activity. This is known as max pooling [11], and it enables a gnostic set to measure the similarity of a pattern to previously observed variants from the object category. This approach differs significantly from the distributed representations that are often used in neural networks [5], [20], since the categorical processing is segregated into distinct sub-networks early in the model.

The gnostic sets also compete with each other, with the least active sets being suppressed. While some of the ideas in my implementation have been independently explored in recent models of object recognition, the role Konorski posits for competition among gnostic sets has not been explicitly modeled. Having gnostic sets compete helps cope with one of the main criticisms of grandmother cell theories, which is that the brain would need an enormous population of neurons devoted to encoding all possible variations of a given category [5], [21]. With competitive normalization, a gnostic unit only partially matching the stimulus will suffice if the units in the competing gnostic sets are less active.

Konorski did not specify that the gnostic units and sets should process data spatially or temporally locally. For aural and olfactory data, this approach is neurally reasonable due to the temporal nature of the data. The relatively small size of IT receptive fields (11 degrees) when objects in natural images are viewed [22] suggests that spatially localized processing may underly object processing in vision as well. In my implementation, the evidence from gnostic sets is spatially or temporally accumulated after competitive normalization of the gnostic sets, simulating one of the roles of later processing in prefrontal brain regions [23], [24].

All of the accumulated evidence from all channels and categories projects to a layer of linear units, which make the final categorization decision. An alternative winner-take-all classification scheme was also explored in experiments. Specific details of the implementation are given in the Materials and Methods section.

## Results

Statistical performance measures consistent with those used in computer vision, machine olfaction, and machine audition were used in my analysis. All of the data sets can be found online and are available for research use.

### Sound Classification

**Figure 2:**
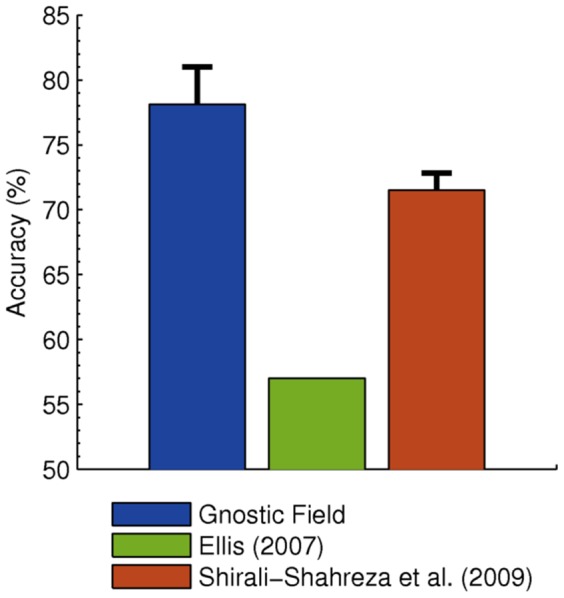
Sound classification results. Accuracy is reported using the official standard for the Artist-20 music database [26], i.e., by averaging results from the six official train/test folds. Guessing the most common category would yield 6% accuracy. Gnostic fields surpass the state-of-the-art model of Shirali-Shahreza et al. [27].

For sound recognition, I used cochleagram features [25], which model the neural firing rate of the human inner ear's basilar membrane. These features were used to train a gnostic field for musical artist classification using the Artist-20 data set [26], which contains music from 20 contemporary artists (e.g., Aerosmith, Queen, Green Day) with 6 albums each (1,413 tracks total). It has six official training and test partitions, which each involve training on five albums per artist and testing on the remaining album. On Artist-20, gnostic fields exceeded the state-of-the-art method, which generates compact signatures for each music track and compares them using bipartite graph matching [27]. These results are given in Fig. 2.

### Odor Classification

**Figure 3:**
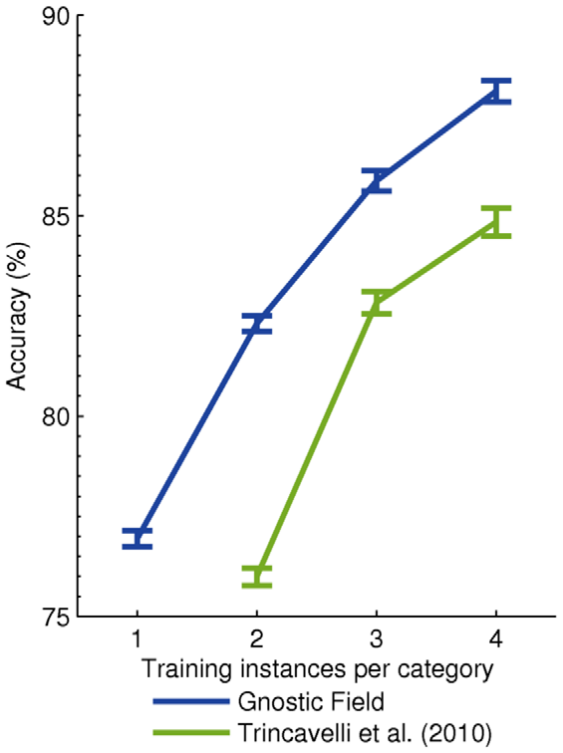
Odor classification results. The plot shows the mean per-category percent accuracy as a function of the number of training instances per category. Chance is 0.93%. Because no classification results exist for this dataset, I implemented the method proposed by Trincavelli et al. [29], one of the best machine olfaction systems. Gnostic fields performed well.

To test the model's performance on olfactory data, I used an e-nose data set [28] consisting of over 100 odorants such as acetone, cyclohexanol, and orange oil. I used the first 500 samples (10 s) of odor exposure and restricted my experiment to substances with five or more instances, leaving 108 categories. I conducted classification experiments on this data set using 50 random splits, with the number of training instances per category varied from 1 to 4 and the remaining data used for evaluation. Gnostic fields performed well compared to one of the best approaches [29], as shown in Fig. 3.

### Object Recognition in Images

For image appearance features, I used dense CSIFT (Colored Scale Invariant Feature Transform) features, which contain pooled histograms of oriented edges from local regions of the image extracted from a luminance channel and two opponent color channels [30].

**Figure 4:**
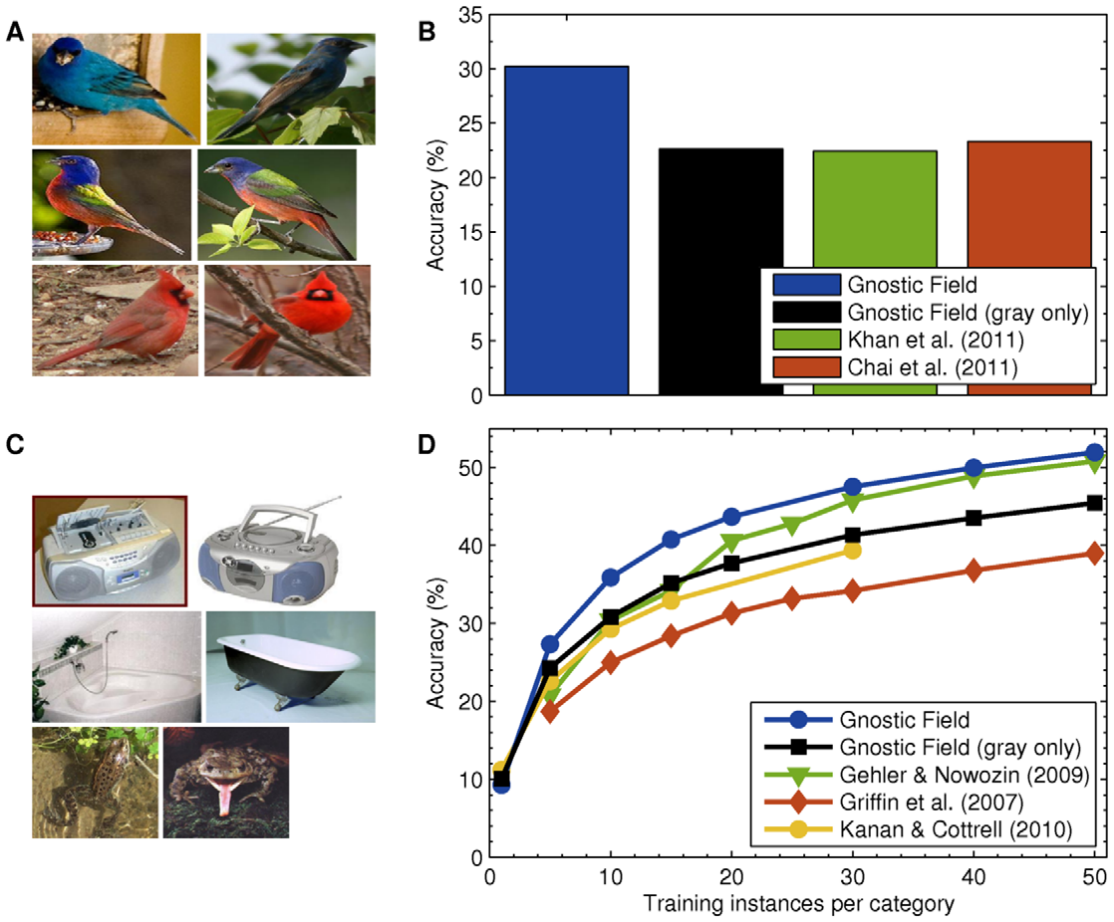
Image classification results. (A) Two exemplar bird images from 3 of the 200 species in CUB-200 [32]. (B) Mean per-class accuracy on CUB-200 when using the official train/test partition, which uses 15 training images per category. Chance is 0.5%. Error bars cannot be computed since there is only a single train/test partition. Gnostic fields achieve high performance compared to state-of-the-art methods, which each combine multiple types of color and grayscale features, including CSIFT. (C) Two exemplar objects images from 3 of the 256 Caltech-256 categories. Like CUB-200, its categories exhibit a great range of intra-class shape and appearance variability. (D) Mean per-class accuracy on Caltech-256 [31] as a function of training instances per category, averaged over 5 train/test folds. Chance is 0.39%. The standard errors for gnostic fields are less than one in all cases. Griffin et al. [31] provide baseline results using grayscale SIFT and spatial pyramid matching. The current state-of-the-art model is Gehler and Nowozin's algorithm [33], which combines 39 kernels using 5 types of engineered gray and color image features. The model of Kanan and Cottrell [34] is among the best methods using a single feature type. It used simulated eye movements and a model of early visual cortex.

I evaluated the model's image recognition abilities on the Caltech-256 [31] and Caltech-UCSD Birds (CUB-200) [32] data sets. For both data sets, the images are weakly labeled, i.e., not segmented and the target object is in its natural background. CUB-200 contains 200 bird species, primarily from North America, and example images are shown in Fig. 4A. Caltech-256 contains 256 general object categories, and the data set is widely used in computer vision. Example Caltech-256 images are shown in Fig. 4C. Results using gnostic fields compared to state-of-the-art methods in computer vision [33]–[36] are given in Figs. 4B and 4D. Almost all of the comparison approaches use many more feature-types extracted at multiple scales [33], [35], [36], whereas only a single feature type is used here. Despite this, gnostic fields exceeded the state-of-the-art approaches.

### The Role of Gnostic Set Competition

Prior to evidence pooling across time or space, gnostic sets compete with each other, with weakly active sets being suppressed by the activity of the most active set. I implemented this using a kind of soft-competitive normalization. In contrast, a more grandmother cell-like scheme would perform a kind of hard-normalization, in which all sets are suppressed except for the most active one. When this was done performance dropped by 4.96% for CUB-200 and by 10.58
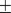
1.04% for Caltech-256 with 15 training instances per category. The change in accuracy was minor for sounds and smells, with performance in both cases being no more than 0.2% worse. This may be because the images contain more noise due to their backgrounds, and grandmother cell coding obliterates useful information from gnostic sets that are only slightly less active than the winning category's set.

### The Role of Whitening

The original (pre-whitened) features could have dimensions that have low variability, but are highly discriminative. This also means that feature dimensions that are highly variable could dominate decision making, even if they are not discriminative. Whitening decorrelates the features and equalizes their variance [16], which mitigates this problem.

I performed experiments to assess the impact of whitening, and for all three modalities removing whitening impaired performance. For images, performance was reduced on CUB-200 by 6.17%. An even greater impairment was observed for sounds, with performance dropping on Artist-20 by 15.26%. Olfactory performance dropped by 3.25% when four training instances per category were used.

### Choice of Classifier

In the main results, I used the Balanced Winnow [37] algorithm to learn the linear output classification weights, which are applied to the normalized and pooled evidence that has been acquired over time or space. However, any linear classification algorithm could potentially be used, and the most popular approach in machine learning is the Support Vector Machine (SVM). SVM's endeavor to maximize the margin between category decision boundaries. Using a multi-category linear SVM algorithm [38] to learn the linear classification weights produced similar results to using Balanced Winnow. With the SVM approach, performance was no more than 1.05% worse across modalities (0.55% for CUB-200, 1.05% for odors, and 0.58% for Artist-20). While Balanced Winnow achieved only slightly better performance, it also has the advantages of using learning rules that are more biologically plausible and being easier to implement (see Materials and Methods).

Using a linear classifier allows information across categories and channels to be combined, and helps defend against similar categories being confused. To test this, I conducted experiments using a single channel with a winner-take-all rule instead of using the linear classification weights. For grayscale features, this resulted in performance dropping on CUB-200 by 2.86%. For sounds, performance was reduced by 6.87%. Finally, for odors performance dropped 5.44% when four training instances were used per category.

### Comparison to HMAX

Some of the mechanisms employed here have been heavily investigated in vision, most notably in the Hierarchical Max (HMAX) framework [11], [39], which provides a model for the primate ventral “object recognition” stream. Like HMAX, gnostic fields use a max-pooling operation; however, in HMAX this is done over features with similar properties (e.g., the same orientation), whereas for gnostic fields pooling is done over features from the same category. Category-specific processing in HMAX occurs primarily at the highest level. Gnostic sets do category-specific processing in local regions of the visual field, with evidence from across the visual field then combined.

I conducted an experiment to compare visual gnostic fields to HMAX. For Caltech-101 [40], a data set that is similar to Caltech-256 [31] but containing 101 categories, one of the best HMAX implementations achieved 51% percent accuracy using 15 training images per category with an SVM classifier [39]. Under the same conditions, a gnostic field achieved 71.41
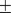
0.40% accuracy with grayscale features and 75.93
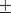
0.44% accuracy with color features. It is possible to use HMAX with a gnostic field, as discussed below.

## Discussion

Konorski proposed a universal theory for recognition across sensory modalities [2]. In this paper, I transformed his theory into a computational model to explore its efficacy at recognizing stimuli. The approach was compared against the best methods in three distinct niches of machine perception, and it achieved state-of-the-art performance. This required filling in the missing details and making modeling decisions to instantiate a version of Konorski's theory. My results indicate that no single component of the implemented framework is solely responsible for the architecture's effectiveness, since when each component is removed performance was impaired in one or more modalities.

One of the appealing aspects of gnostic fields is that there are a multitude of ways to improve and extend them. The easiest way to increase accuracy would be to use additional feature types, which could be incorporated as additional channels. For example, the S2 features produced by HMAX could be used with a visual gnostic field. Several other avenues for improving performance are discussed below.

Gnostic fields are complementary to recent developments in self-taught (unsupervised) feature learning [41], in which unsupervised learning algorithms are used to acquire features that are good for recognition. This approach has been demonstrated to be effective for both auditory and visual data. Self-taught learning could be readily adapted to replace the features used as input to a gnostic field. Deep belief networks [20], an approach closely related to self-taught learning, have also demonstrated good performance on stimuli from different modalities. A key difference between the two approaches is that deep belief networks have a fine tuning step in which all layers of the model are trained, and this is not necessarily true of self-taught learning methods. This allows the low-level features themselves to change with learning, and it would be interesting to explore how this approach could be used with a gnostic field in future work.

My implementation of gnostic fields requires labeled data, but humans and animals are capable of discovering categories in an unsupervised manner. It may be possible to enable unsupervised discovery of gnostic sets by adapting elements of the model given by Waydo and Koch [42]. They used HMAX image features as input to an unsupervised neural network that employed sparse coding principles to learn a representation in which few of the output neurons were active. When they trained the network on a small data set of faces for an identification task, the units learned by the network were selective for particular individuals. They were able to achieve high accuracy on the dataset when they used an SVM-based classifier on the output of their neural network. The max pooling mechanism could potentially be implemented by generalizing their approach to incorporate an additional layer of units.

Gnostic fields gain view robustness by pooling units tuned to coarsely encoded templates learned from previous exposure to individual views of objects. Some have argued that this scheme is not computationally tractable or neurally plausible because an exponential number of units would be needed for such a scheme to be effective [21]. Gnostic fields serve as a counter example, since it is not necessary to represent every possible view and in my implementation. As a function of training data (experience), only a sublinear (polylogarithmic) number of exemplars were learned for each gnostic set (see Materials and Methods). Competitive normalization allows this representation to be efficient, but more work is needed to determine the best form this normalization should take.

While soft-competitive normalization between gnostic sets was vital to achieving high performance on images, it had little impact on sounds and odors when hard competition was used instead. Parameterizing the strength of the competition and making it learnable or context dependent may improve performance across modalities. A potential way to do this would be to fuse gnostic fields with a variant of deep belief networks [20]. This could be done by restricting connectivity in a deep belief network and including competitive subnetworks for each category. This is another approach that could potentially be extended to model unsupervised or semi-superivsed self-organization of gnostic fields from unlabeled stimuli.

Gnostic fields can be implemented with only a preliminary background in machine learning, yielding good “off-the-shelf” performance, with no meta-parameters to adjust. If classification, clustering, and feature extraction toolboxes are available then the algorithm can be implemented in a few hours.

## Materials and Methods

### Input and Whitening

A gnostic field's multidimensional input is segregated into 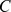 distinct channels. A stimulus from channel 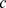 consists of 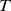 vectors of 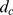-dimensional information, 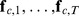. For visual information this consists of composite features from spatial locations in the visual field, for aural data it is distinctive frequency data acquired within a temporal window, and for odors it is the response over time of an array of e-nose sensors.

Using the training data from all categories, the mean 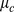 is computed and subtracted from the channel's input. A whitening matrix 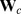 is then learned from the channel's training data using principal component analysis whitening [16], i.e.,
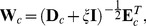
(1)where 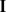 is the identity matrix, 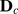 is the diagonal matrix of eigenvalues, 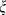 is a regularization parameter (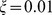 in experiments), and the columns of the matrix 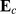 contain the eigenvectors. All principal components are used. Subsequently, the input is normalized to be spherical (unit length), i.e.,
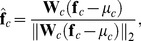
(2)which enables measurements of similarity using dot products [43].

In the experiments that omitted whitening to assess its impact on recognition performance, I set 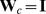.

### Learning Gnostic Units

Gnostic units perform coarse template matching in localized regions of space or time. The activity of a gnostic unit 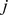 from channel 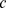, responding to some stimulus from category 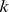 is given by the dot product
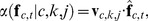
(3)where 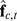 is an 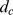-dimensional vector of normalized features encoding the current stimulus at temporal or spatial location 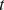, and 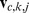 is the neuron's 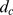-dimensional weight vector. The output of the gnostic set for category 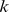 and channel 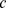 is given by the unit with the largest activity:
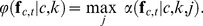
(4)Max pooling allows the gnostic set to respond strongly to any stimuli that matches previously observed variants of the object category [11].

The spherical 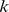-means [44] unsupervised clustering algorithm is used to learn the localized 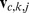 units for each of the 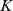 categories and 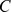 channels. This is done by clustering the whitened training features for each category and channel individually (spherical 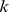-means is run 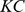 times). I initialized spherical 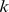-means to a subset of the training data using a variant of the 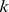-means++ algorithm [45]. Learning gnostic sets in this manner is similar to using clustering to learn the units in a radial basis function neural network [46]. The primary difference between the two approaches is that the input to a gnostic set is spatially or temporally local and the output of the gnostic sets is competitively normalized and then pooled.

Konorski suggested that the number of gnostic units representing a category would depend on the complexity of the modality (dimensionality) and the amount of experience with that category, albeit with fewer units being recruited with increasing exposure [2]. To implement this, the number of 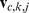 units learned for a category 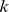 from channel 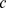 is given by
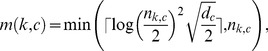
(5)where 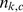 is the total number of that category's feature vectors used for training and 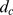 is their dimensionality. None of these parameters are directly tunable, since they depend entirely on the features. The function is polylogarithmic in the number of training feature vectors. This means that with a moderate amount of exposure to a category, 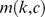 will allocate a relatively small number of units to the gnostic set compared to the number of training observations. For example, the average number of allocated nodes was 0.06% of the total number of training feature vectors for Artist-20 and 2.76% for Caltech-256 with 50 training images per category. However, if very few training observations are available, e.g., one per category, then the number of units allocated will be similar to the number of training instances.

### Competitive Normalization

If multiple gnostic sets are sensitive to the same stimulus, then inhibitive competition suppresses the responses of the least active sets [2]. To implement this for the 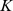 gnostic sets in channel 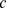, all units have their activity attenuated using
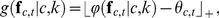
(6)with the threshold 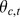 being equal to the population mean, i.e., 
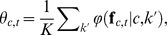
 and 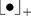 denoting half-wave rectification [47], i.e., setting the negative values to zero. The non-zero responses are normalized using
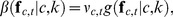
(7)with
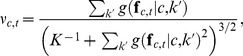
(8)performing a form of divisive normalization that also alters the activity of the gnostic sets according to the population's variability. This is because as the number of categories 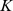 increases, 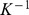 approaches zero, which gives
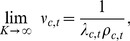
(9)where 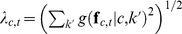 is a divisive normalization term and
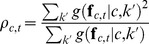
(10)is the contraharmonic mean. The contraharmonic mean is equal to the sum of the arithmetic mean and the Fano factor, the variance divided by the mean, which is a common measure of neural variability. The inverse of the Fano factor is related to the signal-to-noise ratio and thus reweights activity to inhibit the effects of noisier signals.

### Evidence Accumulation and Classification

For each channel, categorical evidence is accumulated, either spatially across the visual field or over time for aural and olfactory stimuli, by summing over and normalizing the activity of the local population representing each class
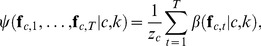
(11)where 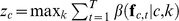, which normalizes activity by the most active category. Note that for some linear classifiers an alternative normalization scheme for 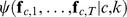 may be advantageous, such as subtracting the mean and dividing by the Euclidean norm.

A linear multi-category classifier decodes the activity of these pooling units. This allows less discriminative channels to be down weighted and it helps the model cope with confused categories. The model's predicted category is given by
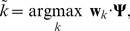
(12)where 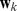 is the weight vector for category 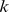 and 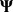 is the response from the 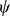 pooling units across all categories and channels combined into a single vector.

In the main results, the 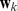 vectors were learned using the Balanced Winnow algorithm [37]. Unlike the Perceptron algorithm, which uses an additive update rule, Winnow instead uses multiplicative updates. Dendritic spines of pyramidal neurons undergo multiplicative changes in size, which suggests that multiplicative learning rules may underlie neuronal learning and memory [48]. To use Balanced Winnow with multiple categories, the linear machine approach was adopted during training, i.e., as each training instance was observed, the weight vector from the correct category was strengthened and the weight vector corresponding to the incorrect category that responded the most was weakened. Balanced Winnow maintains populations of excitatory and inhibitory weights and uses multiplicative updates; however, it is possible to combine these weights into a single representation using the hyperbolic sine (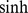) function (see [37] for details). This also results in the learning rules becoming additive in the transformed space. Using the re-expressed version, the learning rules can be extended to the multi-category setting as follows.

For a training instance 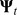 from category 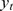, the response of the unit for category 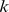 is given by
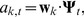
(13)where 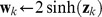 with 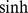 applied to all elements of the vector 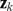. For the correct category 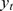, the 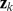 weights are strengthened using
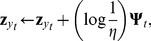
(14)and the weights for the most active incorrect category are weakened by
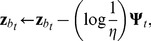
(15)where
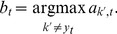
(16)The weight vectors for all other categories are left unaltered. The learning rate 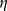 was set to 0.8, and the classifier was trained until the weight vectors converged. Since online learning was unnecessary for this work, a batch version of the algorithm was used in practice.

Experiments were also performed using an SVM to learn the 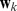 weight vectors. These experiments used the multi-category linear support vector machine (SVM) algorithm of Crammer and Singer [38] from the LIBLINEAR toolbox [49] to learn the linear classification weights, with the cost parameter set low (to 0.0001) since the normalization procedure makes the training vectors very separable.

In the experiments that omitted using the linear classification weights, the decision rule for a single channel was instead given by
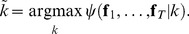
(17)


### Sound Recognition Details

Sounds were recognized using cochleagram features [25], which model basilar membrane neurons. Standard settings were used with Ma's implementation [50] to convert sounds into 48-dimensional vectors containing a sound's constituent frequencies between 50 Hz and 16 kHz, comparable to human hearing. This signal was logarithmically compressed. The interval between successive frames was 10 ms, with a temporal integration time of 8 ms. These settings produce 100 48-dimensional cochleagram features per second.

### Odor Recognition Details

Odor recognition performance was assessed using the largest publicly available e-nose database, which is available in the supplementary materials of [28]. The MOSES II e-nose used to create the data set produces a 16-dimensional time varying signal that I normalized to unit length. Each odor was sampled at 50 Hz, and 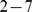 recordings of each odor are available.

Because no classification results exist for it, I implemented the method of Trincavelli et al. [29]. They transform the 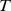 e-nose features from a stimulus into a single vector, which is used with a radial basis function SVM classifier. SVM parameters are tuned using five-fold cross-validation with the training instances per category varied from two to four (cross-validation needs two or more training instances per category).

### Image Recognition Details

Each input image is resized to make its smallest dimension 128 pixels, with the other dimension chosen to preserve the image's aspect ratio. Gamma correction was left intact (see [51] for a discussion of the impact of gamma correction when using SIFT descriptors). Dense CSIFT was configured to use 11
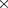
11 spatial bins with a step size (i.e., stride) of 5 pixels, and the dense CSIFT implementation in the VLFeat toolbox was used [52]. While these settings do make the spatial extent of the input relatively small, this is somewhat analogous to the size of receptive fields in IT, which have been shown to be only about 11 degrees of visual angle on average when natural scenes are viewed [22].

Under these settings, about 500–700 high dimensional feature vectors at different locations in the visual field are produced for each of the three image channels, at a single scale. Computing them requires 60 ms (20 ms per channel) on an Intel Core i7-980X in MATLAB R2012a. Only a single scale was used in experiments, but additional scales could be incorporated as extra channels.

Topological information was incorporated into the image features. Let 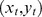 be the location of the 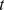
'th feature vector, with these coordinates being normalized by the image size to be between −1 and 1. This was used to construct the location information vector 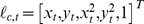, which was normalized to unit length and appended to the 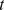
'th feature vector.

Each CUB-200 category has 20–39 images, and the official evaluation set uses 15 training images per category. Images were cropped in the standard manner using the bounding box annotations (see [36]).

For Caltech-256, the model's performance was evaluated using randomly generated train/test partitions, with the number of test images per category fixed at 25 and the number of training images per category varied, mirroring the setup of others [33]. Five partitions were used for each number of training instances, with the mean-per-class accuracy of each partition being reported in Fig. 4D (the standard approach). The same setup was used in the experiments with Caltech-101.
